# The miRNA and PD-1/PD-L1 signaling axis: an arsenal of immunotherapeutic targets against lung cancer

**DOI:** 10.1038/s41420-024-02182-1

**Published:** 2024-09-29

**Authors:** Ritu Yadav, Rinku Khatkar, Kenneth C-H Yap, Chloe Yun-Hui Kang, Juncheng Lyu, Rahul Kumar Singh, Surojit Mandal, Adrija Mohanta, Hiu Yan Lam, Elena Okina, Rajiv Ranjan Kumar, Vivek Uttam, Uttam Sharma, Manju Jain, Hridayesh Prakash, Hardeep Singh Tuli, Alan Prem Kumar, Aklank Jain

**Affiliations:** 1 Non-Coding RNA and Cancer Biology Laboratory, Department of Zoology, Central University of Punjab, Bathinda, Punjab India; 2 Department of Pharmacology, Yong Loo Lin School of Medicine, National University of Singapore, Singapore, Singapore; 3 NUS Centre for Cancer Research (N2CR), Yong Loo Lin School of Medicine, National University of Singapore, Singapore, Singapore; 4 Department of Biochemistry, Central University of Punjab, Bathinda, Punjab India; 5 Amity Centre for Translational Research, Noida, UP India; 6 Department of Biotechnology, MMDU, Ambala, India

**Keywords:** Biomarkers, Cancer

## Abstract

Lung cancer is a severe challenge to the health care system with intrinsic resistance to first and second-line chemo/radiotherapies. In view of the sterile environment of lung cancer, several immunotherapeutic drugs including nivolumab, pembrolizumab, atezolizumab, and durvalumab are currently being used in clinics globally with the intention of releasing exhausted T-cells back against refractory tumor cells. Immunotherapies have a limited response rate and may cause immune-related adverse events (irAEs) in some patients. Hence, a deeper understanding of regulating immune checkpoint interactions could significantly enhance lung cancer treatments. In this review, we explore the role of miRNAs in modulating immunogenic responses against tumors. We discuss various aspects of how manipulating these checkpoints can bias the immune system’s response against lung cancer. Specifically, we examine how altering the miRNA profile can impact the activity of various immune checkpoint inhibitors, focusing on the PD-1/PD-L1 pathway within the complex landscape of lung cancer. We believe that a clear understanding of the host’s miRNA profile can influence the efficacy of checkpoint inhibitors and significantly contribute to existing immunotherapies for lung cancer patients. Additionally, we discuss ongoing clinical trials involving immunotherapeutic drugs, both as standalone treatments and in combination with other therapies, intending to advance the development of immunotherapy for lung cancer.

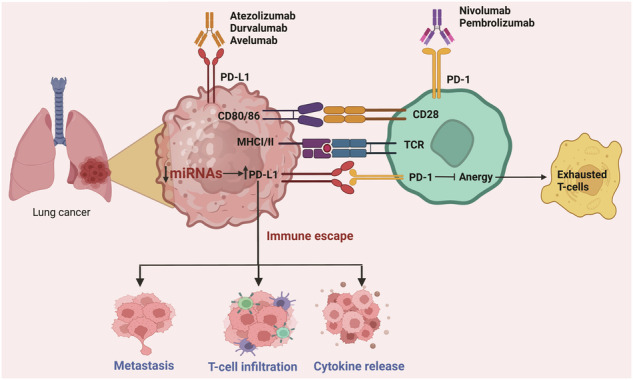

## FACTS

- Immune epigenetic programming of the host limits the outcome of cancer-directed interventions.
- miRNAs landscape drives sterile inflammatory responses in cancer patients.
- miRNAs landscape influences the sensitivity of cancer patients to Immune checkpoint blockade therapy.
- Adjusting microRNAs for immune checkpoint blockade therapy is particularly beneficial in resistant cancers, such as non-small cell lung cancer (NSCLC).

## OPEN QUESTIONS

- How does targeting miRNA overcome the inherent resistance of NSCLC to first and second-line chemotherapy and radiotherapy and enhance the effectiveness of these treatments?
- How modifying the miRNA profile of patients could alleviate T-cell exhaustion in NSCLC?
- How deeper understanding of regulating immune checkpoint interactions significantly enhance lung cancer treatments?
- How altering the miRNA profile in NSCLC patients can increase their sensitivity to various immune checkpoint inhibitors, potentially enhancing the effectiveness of combined chemo-immune therapies by modulating the immune environment and tumor behavior?

## Introduction

Despite early detection and treatment advances in surgery, chemotherapy, radiation therapy, and targeted therapy, the 5-year survival rate for lung cancer patients remains low [1–3]. Unfortunately, with these treatment methods, there is still a significant chance of cancer recurrence with side effects and limited efficacy in advanced cancer stages [4]. Significant progress has been made in the field of immunotherapy for lung cancer and Immune Checkpoint Inhibitors (ICIs) are being increasingly recognized as the favored treatment approach for individuals diagnosed with metastatic, locally advanced, and resectable lung cancer.

**Fig. 1:**
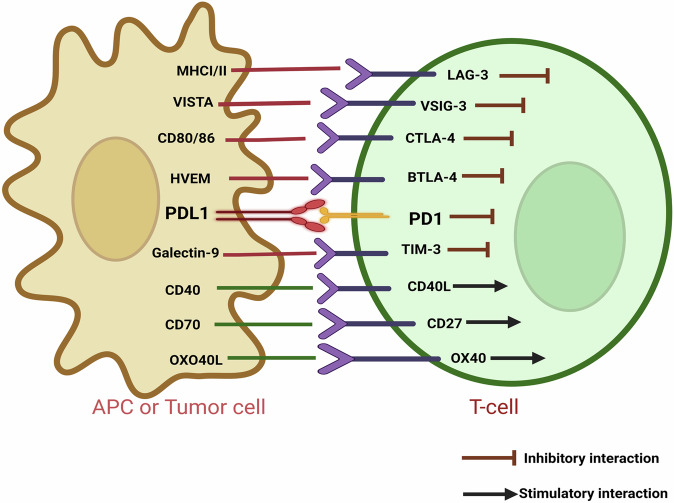
Schematic representation of co-stimulatory and co-inhibitory interactions of APC or Tumor cell and T-cell. The  shows the inhibition of T-cell activation and  symbolizes process of T-cell activation.

Immunological checkpoints are pivotal immune controllers for preserving immune homeostasis and avoiding autoimmune diseases [5, 6]. These checkpoints can either stimulate or inhibit immunological pathways essential for immune self-tolerance and modulating the immune response’s type, intensity, and potency [7]. Some of the checkpoints that have received significant research attention include PD-1 (programmed cell death-1), PD-L1 (programmed cell death ligand-1), CTLA-4 (cytotoxic T-lymphocyte-associated antigen-4), TIM-3 (T-cell immunoglobulin and mucin domain-containing protein-3), LAG-3/CD223 (lymphocyte activation gene-3), VISTA (V-domain immunoglobulin suppressor of T-cell activation), TIGIT (T-cell immunoreceptor with immunoglobulin and immunoreceptor tyrosine-based inhibitory motif domains), B7-H3 (CD276), BTLA (B and T-lymphocyte attenuator), etc. [6]. These immune checkpoints are upregulated in various cancers like lung, breast and bone cancers, as well as melanomas [8]. Optimizing co-stimulatory and co-inhibitory pathways may improve T-cell mediated anti-tumor immune response and cancer immunotherapy as shown in Fig. 1.

Mechanistically, immunotherapeutic drugs such as Ipilimumab (Yervoy), Nivolumab (Opdivo), Pembrolizumab (Keytruda), Cemiplimab (Libtayo), Atezolizumab (Tecentriq) block the interaction between immune checkpoints and their targets. This article focuses on the interference of the PD-1/PD-L1 pathway which prevents host immune evasion by tumor cells, improving anti-tumor immunity [9, 10]. (The detailed mechanism of drug action is described in Section “Role of PD-1/PD-L1 immune checkpoint inhibitors in lung cancer”). ICIs block PD-1 and PD-L1 binding, allowing T-cells to recognize and kill cancer cells. These ICIs have drawn significant interest recently because of their ability to impede co-inhibitory immune pathways during immunotherapy. Studies have shown that these inhibitors improve survival outcomes in patients with advanced-stage lung cancer compared to traditional chemotherapy [11]. Increasing evidence indicates that lung cancer cells with high PD-L1 expression manage to evade immune monitoring by inducing the death of T-cells. [12]. Hence, the immune system becomes unable to distinguish between cancerous and healthy cells [13].

Even though immunotherapeutic drugs have shown promising initial results, the number of patients responding to them is low indicating a need for greater drug development efforts [14]. In this regard, non-coding RNA, such as miRNA, emerges as a promising immunotherapeutic adjuvant. miRNA, having ~22 nucleotides, is a non-coding RNA regulating various biological processes like cell growth, immune surveillance, immunological homeostasis development, and metabolism [15]. miRNA targets mRNAs and suppress protein expression. Aberrant expression of miRNAs can lead to the development of cancer, as they can act as either oncogenes or tumor suppressors. [16]. For example, there is evidence that miR-15/16 are downregulated in a variety of solid cancers, including melanoma, bladder cancer, colorectal cancer, pituitary adenomas, and prostate carcinoma [17]. Additionally, miR-411-5p accelerated the growth of lung tumors, significantly reduced the expression of SPRY4, and simultaneously activates EGFR, AKT signaling, and the epithelial-mesenchymal transition (EMT) in tumor tissues in vivo [18]. A rising corpus of research suggests that immune modulatory miRNAs derived from cancer would also make desirable targets for preventing cancer immune escape [19]. The overexpression of miR-9 in certain malignancies, including glioma, NSCLC (non-small-cell lung cancer), and cervical cancer, can be a determinant factor for a tumor microenvironment with increased immunological tolerance [19]. Also, miR-197 suppresses the cyclin-dependent kinase (CDK), CKS1B/STAT3 signaling axis, hence indirectly inhibiting production of PD-L1. Downregulation of miR-197 is linked with poor overall survival and chemoresistance in a number of malignancies, including lung, pancreatic, and thyroid tumors [20]. Research into miRNA’s roles in the tumor microenvironment has led to the notion that miRNAs could be extremely beneficial in immunotherapy [16]. Recently, our group has also demonstrated that miRNAs 590-5p and 320a act as tumor suppressors by regulating key immunological signaling pathways in lung cancer pathogenesis [21, 22].

Therefore, this review article explores the significance of miRNAs and the PD-1/PD-L1 axis in the context of lung cancer. In particular, the paper will elaborate on the impact of miRNA dysregulation on PD-1/PD-L1 interactions and its effect on lung cancer progression and metastasis. Finally, this paper will review ongoing clinical trials that evaluate the action of immunotherapeutic drugs either as monotherapy or combined with other therapies. Overall, this paper’s analysis aims to support the development of more effective immunotherapeutic drugs against lung cancer.

## Overview of immune checkpoints

The primary focus of this paper is on the PD-1 and PD-L1 checkpoints. However, we will also offer a brief overview of various other important immune checkpoints in the subsequent section. Within this section, we will discuss the structures and functions of key immune checkpoints including PD-1 and PD-L1, particularly in relation to cancer.

### CTLA-4 (Cytotoxic T lymphocyte-associated antigen-4)

**Fig. 2:**
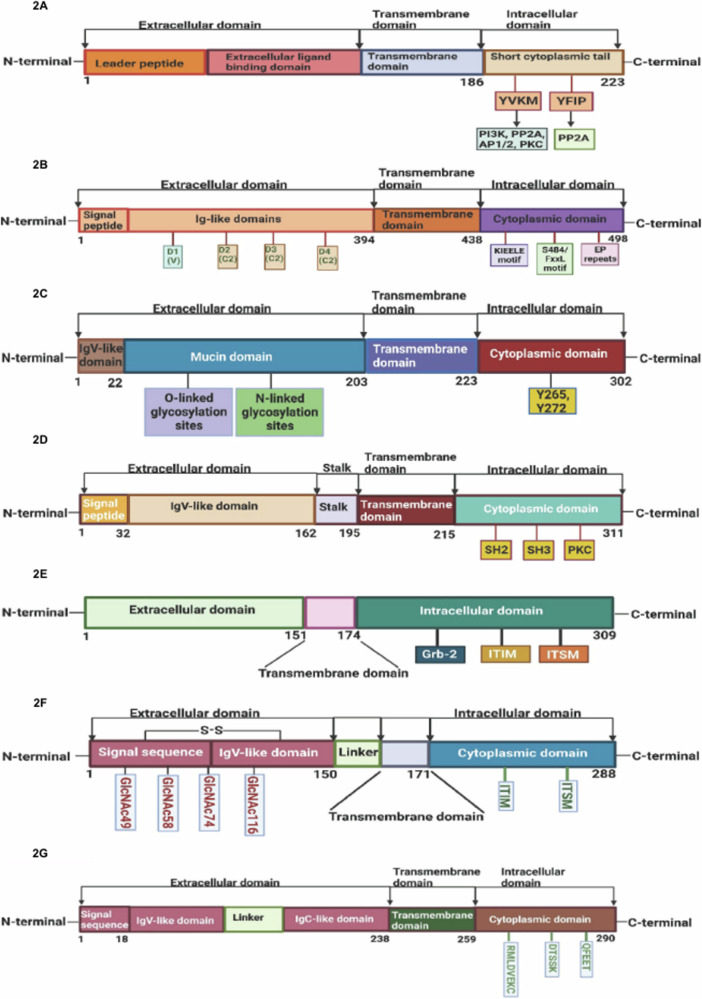
Linear representative structures of various immune checkpoint proteins. Linear structure of CTLA-4 protein consisting of leader peptide and three domains: extracellular ligand binding domain, a transmembrane domain and a short cytoplasmic tail of 37 amino acids having two tyrosine-based motifs. The YVKM motif constitutes a binding site for the lipid PI3K, the phosphatases PP2A, SHP2 and the clathrin adapter protein AP-1 and AP-2. The serine /threonine phosphatases PP2A binds to the lysine-rich motif and tyrosine 218 (**A**). Molecular organization of LAG-3 protein. The domain organization of LAG-3 is schematically shown in the (**B**), with each Ig-like domain are indicated in rectangular boxes where D1 domain represents the variable and D2- D4 shows the constant domains respectively. The cytoplasmic domain has three conserved regions: a serine-phosphorylation site, a KIEELE motif (Lysine Isoleucine Glutamate Leucine Glutamate), and glutamic acid-proline repeats, in which the function of KIEELE motif is still unclear, but the current literature suggests that it is essential for LAG-3 to perform its inhibitory function. Linear representation of TIM-3 protein. It consists of four domains: an IgV-like domain of 22 amino acids, mucin domain of 181 amino acids containing O and N-linked glycosylation sites followed by the cytoplasmic domain of 79 amino acids having tyrosine phosphorylation sites (**C**). Linear representation of the domain structure of VISTA protein encoded by *VSIR* gene. VISTA is composed of 311 amino acids in which N-terminal domain, a signal peptide of 32 amino acids, an IgV-like domain of 130 amino acids, stalk domain of 33 amino acids, transmembrane domain of 20 amino acids and cytoplasmic domain of 96 amino acids respectively possessing SH2, SH3 and PKC domains (**D**). Linear representation of domain structures of BTLA protein encoded by *BTLA* gene. BTLA is composed of 309 amino acids in which ectodomain of 151 amino acids, transmembrane domain of 23 amino acids and cytoplasmic domain of 135 amino acids. The cytoplasmic domain contains Grb-2, ITIM and ITSM motifs responsible for its inhibitory signaling (**E**). Linear representation of the domain structures of PD-1 protein encoded by *PDCD1* gene. PD-1 is composed of 288 amino acids. The extracellular domain contains an N-terminal domain containing a signal sequence and IgV-like domain of about 150 amino acids. Moreover, the signal sequence and IgV-like domain are connected by disulfide bond (C54-C123). The extracellular domain possesses four N-acetylation sites at N49, N58, N74 and N116. The cytoplasmic domain of 117 amino acids having ITIM and ITSM motifs (**F**). Linear representation of the domain structures of PD-L1 protein encoded by *CD274* gene. PD-L1 is composed of 290 amino acids in which N-terminal domain, a signal sequence of 18 amino acids, an IgV-like domain followed by a linker with IgC-like domains counting for 220 amino acids. The transmembrane domain of 21 amino acids and the cytoplasmic domain of 31 amino acids having QFEET, DTSSK and RMLDVEKC motifs (**G**).

CTLA-4 (Cytotoxic T lymphocyte-associated antigen-4) or CD152 (cluster of differentiation 152) is a type 1 transmembrane glycoprotein. It belongs to the Ig superfamily [23] and is crucial to the immune response against cancer cells. It contains 223 amino acids and has a molecular weight of ~33 kDa (Fig. 2A). An extracellular ligand-binding domain, a transmembrane domain, a leader peptide, and a short cytoplasmic tail make up the four domains of the CTLA-4 protein [23].

CTLA-4 is typically expressed on the surface of activated T-cells. However, regulatory T-cells (Tregs) express CTLA-4 ubiquitously because of the high expression of the forkhead transcription factor, FoxP3. FoxP3 positively regulates the expression of CTLA-4 [24, 25]. CTLA-4 works by competing with another T-cell co-stimulatory receptor, such as CD28, to bind to its ligands CD80 (also known as B7-1) and CD86 (also known as B7-2). The CD80 and CD86 are expressed on the surface of antigen-presenting cells [26]. By binding to its ligands, CTLA-4 reduces the level of IL-2 synthesis, which results in the release of inhibitory signals that suppress T-cell activation and proliferation, thus preventing excessive immune responses that could damage healthy tissues [27–29]. In cancer, tumor cells evade the immune system by expressing CTLA-4, allowing the tumor cells to inhibit the immune response and continue growing and spreading throughout the body [30].

Given its role in regulating the immune system, CTLA-4 has become an important target for cancer immunotherapy. For example, ipilimumab has been shown to be a promising immunotherapeutic drug that blocks CTLA-4 and enhances the anti-tumor immune response in advanced melanoma [31].

### LAG-3 (Lymphocyte activation gene-3)

The immune checkpoint LAG-3 (Lymphocyte activation gene-3), CD223 (cluster of differentiation 223) or FDC protein is a type I transmembrane protein with 498 amino acids. A fraction of natural killer (NK) cells, activated CD4^+^ and CD8^+^ T-cells, Tregs, B-cells, and plasmacytoid dendritic cells (pDCs) express LAG-3. [32]. It has a molecular weight of 50 kDa and is comprised of four domains: a hydrophobic leader, an extracellular region, a transmembrane region, and a cytoplasmic region [33] (Fig. 2B). The extracellular region comprises four immunoglobulins (Ig) superfamily-like domains (D1-D4) [34]. The membrane-distal D1 domain contains a unique short sequence of 33 amino acids called an “extra loop.” The cytoplasmic domain has three conserved regions: a serine-phosphorylation site, a KIEELE motif (Lysine Isoleucine Glutamate Leucine Glutamate), and glutamic acid-proline repeats. While the function of KIEELE motif is still unclear, current literature suggests that it is essential for LAG-3 to perform its inhibitory function [35]. On tumor-infiltrating lymphocytes (TILs), LAG-3 and PD-1 are frequently co-expressed and elevated, which causes immune exhaustion and permits immune evasion by cancer cells [7]. MHC-II is typically regarded as a ligand for LAG-3. The binding affinity between LAG-3 and MHC-II is a 100-fold higher than CD4’s binding affinity with MHC-II resulting in the inhibition of T-cell activation with LAG-3 expression [36]. Also, constitutive LAG-3 expression is often associated with exhausted T-cells. It is widely accepted to be a marker of exhaustion for CD4^+^ and CD8^+^ T-cells that have been exposed to repeated antigen stimulation in cancer [37].

LAG-3 has also been observed to bind to TCR in both CD8^+^ and CD4^+^ T-cells leading to the inhibition of TCR-dependent tumor cascades and the curbing of T-cell responses. However, the precise mechanisms of LAG-3’s activity and its connection to other immune checkpoint molecules are still unknown [34]. This is partially attributed to the unconventional signaling motifs in its intracellular domain that vary from other traditional immunoregulatory signaling motifs but have analogous inhibitory properties [32]. In numerous solid tumors such as ovarian cancer, melanoma, colorectal cancer, and hematological malignancies, including Hodgkin and diffuse large B-cell lymphoma, TILs with elevated LAG-3 expression have been identified [32]. Since LAG-3 expression on activated immune cells directly correlates with the inhibitory effect of the protein, blocking and inhibiting LAG-3 expression with antibodies or small molecule inhibitors is critically essential [38]. For example, Retilimab, an antibody specifically targeting LAG-3, has been utilized with the PD-1 inhibitor nivolumab to treat metastatic or unresectable melanoma [39, 40].

### TIM-3 (T-cell immunoglobulin and mucin domain containing protein-3)

The *TIM* gene family comprises three genes, namely TIM-1, TIM-3, and TIM-4. The human TIM-3 protein belongs to the Ig superfamily (IgSF), composed of 302 amino acids [41]. TIM-3, or HAVCR2 (hepatitis A virus cellular receptor 2) [42], is expressed on several types of immune cells, including T-cells, B-cells, Tregs, NK-cells, DCs, monocytes, and macrophages [7, 43]. Extracellular, transmembrane, and intracellular segments make up the TIM-3 protein structure (Fig. 2C). ITIM (immune receptor tyrosine-based inhibitory motif) or ITSM (immune receptor tyrosine-based switch motif) are examples of conventional inhibitory signaling motifs that are absent from the cytoplasmic tail of the TIM-3 [44]. TIM-3 contains five conserved tyrosine residues, Tyrosine residues Y265 and Y272 are particularly important as they play an important role in downstream signaling upon phosphorylation by Src kinases or interleukin-inducible T-cell kinase[41]. The Bat3 protein, also known as HLA-B-associated transcript, has been reported to bind to the cytoplasmic tail of TIM-3 and inhibit signaling function. Specifically, Bat3 prevents the binding of proteins containing SH2 domains, such as tyrosine-protein kinase (Fyn) and lymphocyte-specific protein tyrosine kinase (Lck), to the phosphorylated tyrosine tail of TIM-3, which is required for downstream signaling events [45]. T-cell anergy is thought to be induced by Fyn [46]. It is shown that Tim-3’s cytoplasmic tail interacts with TCR complex elements, and is influenced by the ratio of Bat-3 to Fyn bound to the intracellular tail of Tim-3 [45]. Also, galectin-9 (Gal-9), high mobility group protein B1 (HMGB1), carcinoembryonic antigen cell adhesion molecule-1 (Ceacam-1), and phosphatidylserine ligands are found to interact with TIM-3 domain [41].

It has been demonstrated that there is a substantial amount of TIM-3 present on tumor antigen-specific T-cells in the peripheral blood and among TILs [47]. TIM-3 is generally known as a negative regulator of anti-tumor immunity and low levels of T-cells from peripheral blood have been associated with poor patient survival [41]. Additionally, it has been demonstrated that TIM-3 plays a critical role in inhibiting anti-tumor immunity by mediating T-cell exhaustion. Studies have shown that T-cells expressing both TIM-3 and CD8^+^ T exhibit compromised Stat5 and p38 signaling pathways. Notably, blocking the TIM-3 pathway has been observed to boost cancer immunity, leading to greater production of IFN-γ in T-cells. Consequently, numerous companies are currently conducting clinical trials involving TIM-3 inhibitors due to their potential as a form of cancer treatment.

### VISTA (V-domain Ig suppressor of T-cell activation)

VISTA is a transmembrane protein that belongs to the B7 family of co-stimulatory and co-inhibitory molecules regulating T-cell activation and immune responses [48]. VISTA performs dual roles on T-cells, as a receptor and a ligand produced on antigen-presenting cells. The human VISTA protein comprises 309 amino acids and has a molecular weight of ~34.7 kDa [49]. The extracellular domain of VISTA contains a V-type immunoglobulin domain, necessary for the protein’s interaction with its ligands on T-cells [50] (Fig. 2D). The cytoplasmic domain of VISTA includes a short sequence of amino acids responsible for its intracellular signaling function [51]. A crystal structure analysis of the extracellular domain of VISTA shows a binding site for the ligands CD28 and CD4, on T-cells [52].

Leukocytes, myeloid cells, neutrophils, and microglia, express the most VISTA proteins, followed by monocytes, macrophages, and dendritic cells [53]. Due to the sequence homology of the B7 family of ligands and receptors with human VISTA, a negative immune checkpoint regulator is currently in phase I clinical trials (NCT02671955) [54]. The sequence homology between the therapeutic agent and natural ligands of immune checkpoints enhances the specificity and binding affinity of the therapeutic agent to its target. However, further research is required for anti-VISTA-mediated immunotherapy [55].

### BTLA (B and T- lymphocyte attenuator)

The immune checkpoint molecule BTLA, produced in activated T-helper cells, is a B and T-lymphocyte attenuator protein [56]. The BTLA protein is 309 amino acids long with a molecular weight of about 32 kDa. BTLA belongs to the CD28 protein family, and it has sequences conserved across different species, with over 90% similarity between humans and mice [57]. BTLA is a glycosylated receptor protein that contains an extracellular domain composed of two immunoglobulin-like domains and a transmembrane domain that anchors the protein to the cell membrane [58] (Fig. 2E). In normal conditions, the herpesvirus entry mediator (HVEM) ligand binds to the extracellular domain of BTLA, which helps prevent the immune system from becoming overactive and causing damage to healthy tissues [59]. Lymphocytes highly express BTLA which blocks B and T-cell activation and proliferation, inducing immunosuppression[56]. Moreover, it was found that inhibition by BTLA is stronger than stimulation by HVEM on T-cells, preventing the overactivation of T-cells [60]. The formation of a cis-heterodimeric complex between HVEM and BTLA in I T-cells is crucial for sustaining T-cell tolerance because it prevents external CD160 and other co-signaling molecules from attaching to HVEM and activating the NF-kB signaling pathway [61].

The cytoplasmic domain of BTLA has ITIM which helps recruit proteins that can inhibit signaling pathways. BTLA has been linked to a defective anti-tumor immunological response in various cancers [56]. This can be seen in gallbladder cancer, in which a higher percentage of BTLA^+^ CD8^+^ cells is associated with a poorer prognosis [62]. Additionally, increased BTLA expression has also been found in T-cells from individuals with lung cancer [63].

Recently, BTLA-HVEM interactions have been utilized as a potential target for checkpoint blockades in cancer therapy.

### PD-1 (programmed cell death-1)

Human PD-1, or *CD279*, is a member of the immunoglobulin gene superfamily [64]. It is a cell surface receptor that serves as a T-cell checkpoint [65]. PD-1 is a type I transmembrane protein that weighs ~55 kDa with a length of 288 amino acid residues. It possesses a single IgV extracellular domain [66] along with ITIM and ITSM in the cytoplasmic domain, required for the inhibition of TCR signaling [67] (Fig. 2F). PD-1 is typically expressed on the surface of T-cells, B-cells, dendritic cells, monocytes, and activated natural NK-cells [67]. Several transcription factors, including NFATC1 (nuclear factor of activated T-cells, cytoplasmic 1), FOXO1 (forkhead box protein O1), T-bet (T-box expressed in T-cells) or TBX21 (T-box transcription factor 21), and BLIMP1 (B lymphocyte-induced maturation protein 1) regulate PD-1 expression during antigen-activation [68]. Structurally, PD-1 contains three loops—N-terminal, FG, and BC loops which recognize the nivolumab binding site to PD-1. [69]. The N-terminal extension of PD-1, which is outside of the protein’s Ig-like V-type domain, is the major site via which nivolumab binds to it [70].

There are five known human PD-1 isoforms. In addition to the full-length isoform, the alternatively spliced PD-1 mRNA transcripts (PD-1Δex2, PD-1Δex3, PD-1Δex2,3, and PD-1Δex2,3,4) have also been found [65]. Exon 2 (the extracellular IgV-like domain) and exon 3 (the transmembrane domain) are removed to create PD-1-ex2 and PD-1-ex3 respectively through alternative splicing. Exons 2 and 3 are absent from PD-1-ex2,3. [65]. PD-1Δex2,3,4 does not have exon 2, 3, and 4 (intracellular domain) and contains a premature stop codon in exon 5. Disrupting the PD-1 pathway can result in uncontrolled T-cell responses, autoimmunity, and reduced immune tolerance [71].

PD-1 performs two opposing roles in regulating immune functions. While it is crucial for preserving immune tolerance and reducing harmful or ineffective immune reactions, PD-1 can also disrupt the immune system’s defense mechanisms, allowing cancer cell proliferation [72].

### PD-L1 (programmed cell death ligand-1)

PD-L1 (programmed cell death ligand 1) or CD274 is a type 1 transmembrane glycoprotein. It belongs to the B7 ligand family and encodes 290 amino acids [73]. It is expressed on T-cells, B-cells, dendritic cells, monocytes, macrophages, and some epithelial cells, especially in inflammatory conditions [74, 75]. PD-L1 consists of IgV and IgC structural domains, hydrophobic transmembrane domains, and cytoplasmic tail structure domains (Fig. 2G). It has also been related to an immunological milieu with an increased concentration of CD8^+^ T-cells, generation of Th1 cytokines and chemical agents, interferons, and specific gene expression traits [76].

Studies have shown that various tumor types, including NSCLC, hematological malignancies, and virus-infected cells, express PD-L1 on their surface [77]. It is speculated that the increased synthesis of reactive oxygen species and the activation of NF-kB are responsible for the increased PD-L1 expression. [78]. Additionally, research has indicated that the overexpression of PD-L1 in ovarian cancer cells is linked to disease progression and is a result of IFN-γ produced by CD8^+^ cells. In the mouse model of acute myeloid leukemia (AML), IFN-γ receptor 1 (IFNGR1) suppression can decrease the expression of PD-L1 through the MEK/ERK and MYD88/TRAF6 pathways [79]. Studies on melanoma cells have demonstrated that the PD-L1 gene might be regulated by IFN-γ produced by T-cells through the JAK/STAT/IRF1 (Interferon regulatory factor 1) pathway [80]. Secretion of IFN-γ by T-cells and NK-cells seems to be associated with an increase in PD-L1 on tumor cells. In contrast, blocking of EGFR signaling may decrease the expression of PD-L1, enhancing the cellular immune response against the tumor [81].

## T-cell priming and exhaustion

### T-cell priming

**Fig. 3:**
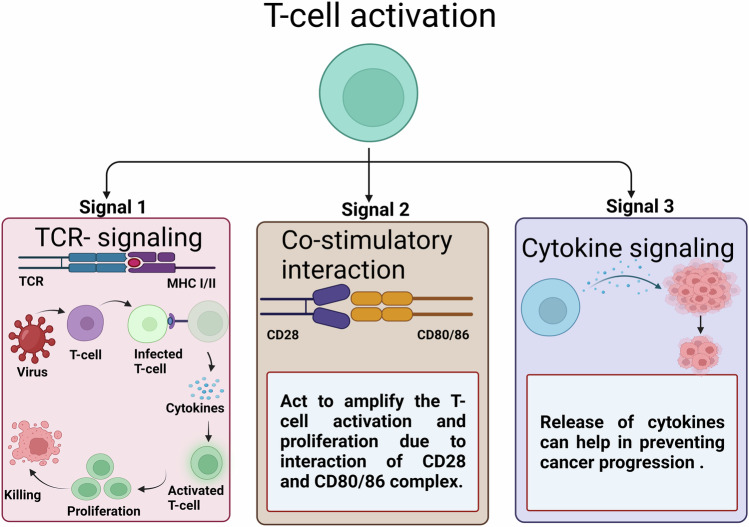
Three signal hypotheses for T-cell activation: Signal 1-TCR-signaling; Signal 2- Co-stimulatory interaction between CD28 and CD80/86; Signal 3- Cytokine signaling.

T-cell priming and cancer immunotherapy are deeply interconnected processes in immuno-oncology. In cancer, the immune system often fails to recognize and target cancer cells as foreign, allowing the tumor to grow and spread unchecked. T-cell priming involves the activation of naive T-cells, transforming them into effector CD8^+^ cytotoxic T-cell lymphocytes (CTLs) capable of targeting specific pathogens or cancer cells [82]. This process typically produces long-lasting and effective anti-tumor immune responses[83]. This priming process typically produces long-lasting and effective anti-tumor immune responses. The priming of CD8^+^ T-cells relies on three key signals: TCR engagement by peptide/MHC complexes (signal 1), co-stimulation by CD28 and CD80/CD86 (signal 2), as well as specific cytokine signaling (signal 3) [84, 85] (Fig. 3). The relative potency of these three signals defines the fate of primed T-cells and the extent of their growth [85]. Cancer cells can express abnormal proteins on their surface, which can be recognized as foreign by the immune system and antigen-presenting cells (APCs) can present these tumor-associated antigens to T-cells, activating them and initiating an immune response against the cancer cells. Once activated, the T-cells can migrate to the tumor site and trigger the destruction of the cancer cells [86]. However, recent reports suggest that cancer-associated fibroblasts (CAFs), macrophage type 2 (M2) cells, and regulatory T-cells (Tregs) form immunologic barriers to CD8^+^ T-cell-mediated anti-tumor immune responses [87]. Additionally, checkpoint inhibitors often block proteins that suppress T-cell activity, thereby maintaining the immune response against cancer cells.

Therefore, the success of immunotherapies depends on effective T-cell priming, as strong and prolonged T-cell activity is essential for removing cancer cells and achieving good treatment results.

### T- cell exhaustion

**Fig. 4:**
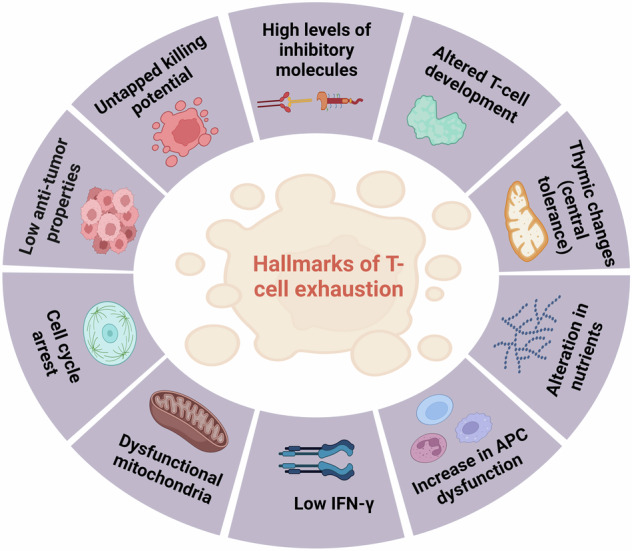
Hallmarks of T-cell exhaustion: It shows low anti-tumor properties, high levels of inhibitory molecules, untapped killing potential, altered use of nutrients, dysfunctional mitochondria, and failure to transition to quiescence and acquire antigen-independent memory T-cell homeostatic responsiveness and many more.

The interaction between the innate and adaptive immune systems is essential to the natural immune response. The surveillance, detection, and extermination of cancer cells are crucial components of anticancer immunity [88]. In the context of cancer, T-cell exhaustion is a phenomenon in which T-cells become unresponsive to the tumor antigens they encounter and are thus unable to carry out an effective immune response against the tumor [89, 90]. It is a kind of T-cell malfunction that occurs throughout several chronic infections and cancers, characterized by poor effector activity, persistent expression of inhibitory receptors, and a transcriptional state different from that of functioning effector or memory T-cells [91]. Exhausted T-cells in the tumor microenvironment exhibit overexpression of inhibitory receptors, reduced effector cytokine secretion, and cytolytic activity, resulting in cancer elimination failure [87]. Several factors contribute to T-cell exhaustion in cancer. One such factor is the interaction of PD-1, CTLA-4, and TIM-3 inhibitory receptors on the T-cell surface with ligands on the surface of the cancer cell, which sends an inhibitory signal to the T-cell to halt its anti-tumor activity, rendering it ineffective against the tumor [92–94]. Exhaustion makes it difficult to handle infections and malignancies effectively. Other factors contributing to T-cell exhaustion include continuous exposure to antigens coupled with high levels of pro-inflammatory cytokines, and metabolic stress within the tumor microenvironment. These factors can collectively lead to T-cell dysfunction and exhaustion (Fig. 4).

Recent research has described T-cell exhaustion in various experimental and clinical contexts, providing a deeper understanding of its functional and phenotypic features. Although the complete mechanisms are still being elucidated, advances in the molecular characterization of T-cell exhaustion are uncovering the underlying causes and pointing to promising therapeutic options [90]. Restoring exhausted T-cells is an innovative cancer treatment technique that has shown encouraging results, marking a significant advance in cancer immunotherapy [95].

## The mechanism of action of PD-1/PD-L1 in T-cell inhibition

As the focus of this paper is PD-1/PD-L1, in this section, we discuss the mechanistic role of PD-1 and PD-L1 in T-cell inhibition.

**Fig. 5:**
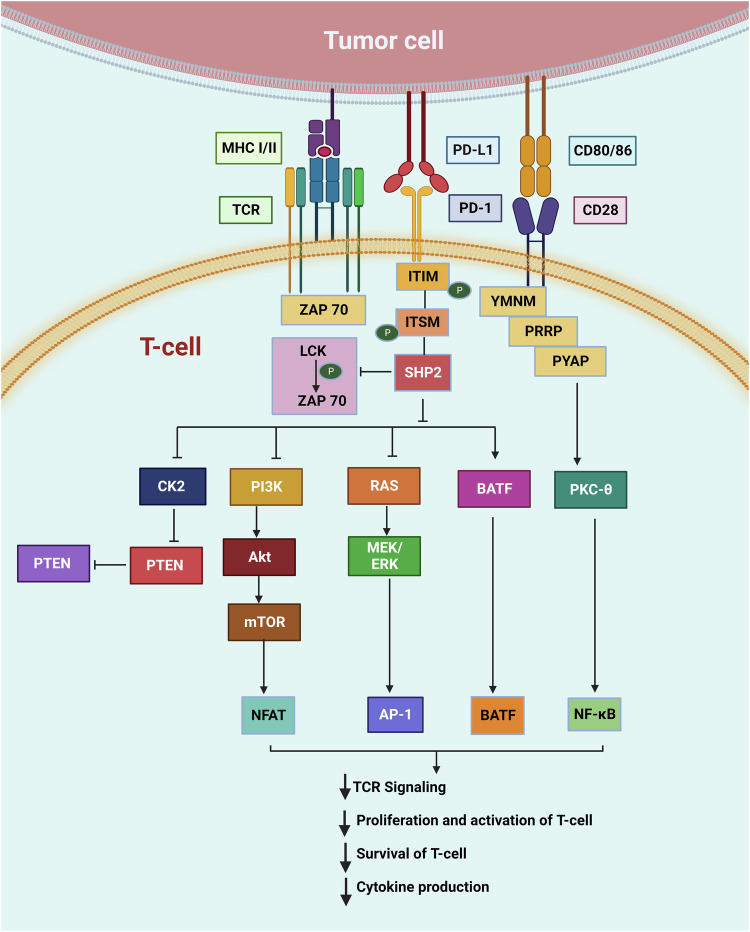
A snapshot of PD-1/PD-L1 signaling pathway in T-cells: When PD-1 is engaged with its ligand PD-L1, the ITIM and ITSM motifs in the cytoplasmic domain of PD-1 get phosphorylated, leading to the recruitment of SHP1/2 proteins. These proteins antagonize the positive signals of T-cell activation, inhibiting T-cell proliferation, growth, and survival.

PD-1 and its ligand PD-L1 are indispensable components in tumor immune evasion, enabling the tumor to remain undetected and survive in the tumor microenvironment [96]. PD1/PD-L1 interaction between tumor cell and T-cells causes T-cell exhaustion and anti-inflammatory interleukin-10 (IL-10) production [64]. Hence, PD-L1 overexpression shields a tumor cell from CD8^+^ T-cell-mediated killing [97]. Additional interactions between PD-L1 and other surface ligands such as CD80 on activated T-cells and APCs further inhibit the activation of T-cells [75]. PD-1/PD-L1 induces the phosphorylation of ITIMs and ITSMs in the cytoplasmic domain of PD-1, and subsequently recruits Src homology phosphatases 1 or 2 (SHP-1/SHP-2) to PD-1 [98]. These phosphatases inhibit signaling pathways downstream of the T-cell antigen receptor (TCR), such as the phosphoinositide 3-kinase (PI3K) and the Ras-MAPK (mitogen-activated protein-kinase) pathways (Fig. 5), thus blocking T-cell activation, proliferation and cytokine production, and ultimately culminating in the loss of immunological functionality [99, 100]. It was previously shown that pharmacological inhibition of PI3K was able to block the IFN-γ-induced upregulation of PD-L1 expression in lung cancer cells [101]. Moreover, pharmacological inhibition of the MAPK pathway was able to block the EGF- and IFN-γ-induced upregulation of PD-L1 in lung cancer cells [102].

## The miRNAs and PD-1/PD-L1 signaling axis

MiRNAs have gained attention because of their extraordinary contribution to tumor development by controlling the various hallmarks of cancer [103]. Their numerous roles in lung cancer as tumor-suppressing, oncogenic, diagnostic, and prognostic biomarkers have been firmly cemented by a huge body of data from multiple studies.

Further, miRNAs have also been implicated in the regulation of PD-1/PD-L1 immune checkpoints [104]. miRNAs play a crucial role in enabling tumor immune evasion, leading to enhanced tumor growth and progression. Therefore, it is essential to investigate the expression of miRNAs and understand their relevance in the context of lung cancer [105]. Understanding the role of miRNAs in regulating the immune checkpoint signaling pathway is vital, as it could reveal novel mechanisms that can be targeted to improve the efficacy of immunotherapy in cancer patients [106]. The various miRNAs that have been implicated in lung cancer are discussed below and have also been summarized in Table 1.

**Table 1:** Various dysregulated miRNAs with respect to PD-1/PD-L1 in lung cancer.

_Sr.No._	_miRNA_	_Regulation_	_Property_	_In-vitro and in-vivo systems used_	_Target_	_Validation methods_	_References_
_1._	_miR-4458_	_downregulated_	_Tumor-suppressor_	_A549, H1299, SW900, LCC (mouse lung cancer cell line), normal human lung fibroblast cell line 2BS; C57BL/6 mice model_	_*STAT3*_	_ELISA, Western blot, CCK-8 assay, Luciferase assay, qRT-PCR_	[113]
_2._	_miR-526b-3p_	_downregulated_	_Tumor-suppressor_	_BEAS-2B, A549, H1975, PC-9; A549 metaststic xenograft models (BALB/c nude mice)_	_*STAT3*_	_miRNA microarray analysis, MTT assay, Migration assay, Western blot, qRT-PCR, Luciferase assay, IHC_	[118]
_3._	_miR-34a_	_downregulated_	_Tumor-suppressor_	_A549, H460, H1299; 344SQ syngeneic mouse model, H1299 xenograft model_	_*PD-L1*_	_IHC, Flow cytometry qRT-PCR, Western blotting, Luciferase assay_	[119]
_4._	_miR-200b_	_downregulated_	_Tumor-suppressor_	_393P, 393LN, 344P, 344SQ, 531LN2, 531LN3, LLC-JSP Lewis lung cell lines; 129/Sv mice_	_*ZEB1*_	_Immunohistochemistry, Flow-cytometry, qRT-PCR, Luciferase assay_	[126]
_5._	_miR-16-5p_	_downregulated_	_Tumor-suppressor_	_A549, PC9, HCC827; BALB/c nude male mice_	_*PD-L1*_	_IHC, Western blot, Colony formation assay, Wound healing assay, Flow cytometry, qRT-PCR_	[130]
_6._	_miR-320a_	_downregulated_	_Tumor-suppressor_	_A/J mice model_	_*PD-L1*_	_IHC, Western blot, In-situ hybridization_	[135]
_7._	_miR-4315_	_upregulated_	_Oncogenic_	_A172, MCF7, OV9, A172, A549; MNRI Nude mice_	_Bim_	_ELISA, Luciferase assay_	[136]
_8._	_miR-181a_	_downregulated_	_Tumor-suppressor_	_A549, H69, LL/2 cells; C57BL6/J mice model_	_*STAT3*_	_qRT-PCR, Western blot, Flow cytometry, MTT assay_	[141]

### miR-4458

miR-4458 is located on chromosome 5p15.31 and has been shown to act as a tumor suppressor in haemangiomas [107], triple-negative breast cancer [108], hepatocellular carcinoma [109] and NSCLC [105]. However, various studies have also reported its differential upregulation in colon cancer [110], and prostate cancer [111]. Moreover, it has been shown to play both oncogenic and tumor-suppressor roles in certain cancers such of the thyroid (as per miRNA sequencing profiling), ovary [112, 113], and of the colon [114]. Although its mechanistic role in cell proliferation and anti-tumor immunity is unclear, some recent studies have demonstrated that miR-4458 blocks cell proliferation [115] and suppresses tumor cell migration [116]. Studies have shown that miR-4458 can modulate immune responses by regulating the expression of various genes involved in immune cell activation and differentiation. For example, miR-4458 has been found to target the expression of the gene encoding the pro-inflammatory cytokine interleukin-6 (IL-6) in dendritic cells, leading to a reduction in IL-6 production and subsequent suppression of T-cell activation.

Regarding the role of miR-4458 in NSCLC immunotherapy, Liu et al. [117] found that in NSCLC patients, PD-L1 expression is elevated, in contrast with decreasing miR-4458 levels, leading to increased cell proliferation and tumor growth [117]. The predicted binding site on PD-L1 mRNA by miR-4458 is shown in Table 2. Furthermore, the authors found a lower proportion of NK-cells, CD8^+^ T-cells, CD4^+^ T-cells, and CD3^+^ T-cells in the peripheral blood of NSCLC patients compared to healthy subjects. Similarly, compared to normal human embryonic lung fibroblast cells (2BS), miR-4458 was found to be downregulated in human NSCLC cell lines (~1.6 fold in H1299, ~1.9 fold in A549 and ~1.4 fold in SW900) (*p* < 0.01), whereas a higher expression level of STAT3 was reported in lung cancer cells (~1.6 fold in H1299, ~1.734 fold in A549, and ~1.75 fold in SW900) (*p* < 0.01). The above data suggests that miR-4458 exists as a tumor suppressor in lung cancer.

**Table 2:** Predicted miRNAs binding sites on PD-L1.

Sr. No.	miRNA	Target	Starting position of predicted target site(s)	Folding energy (kcal/mol)	Binding sites	p-value
1.	miR-4458	PD-L1	742	−16.70	G**TC**a**TCCCA**gaa**CTACCTT** a**AG**a**AGG**t**GT**g**GATGGAGA**	2.01E-01
2.	miR-526b-5p	PD-L1	275; 2009	−14.20; 15.00	**A**tg**GA**g**AGG**aaga**CCT**g**AA**gt **T**gt**CT**t**TC**a**C**gaag**GGA**g**TTC**c g**CA**a**GA**ca**AAGT**ac**CT**gt**CCCAA**ga t**GT**-**CT**--**TTCA**-c**GA**ag**GGAGTTC**tc	4.25E-2,1.86E-2
3.	miR-200b-5p	PD-L1	51	−15.10	tg**CA**gg**GC**att**CCAG**a**AAGATG** ag**GT**ta**CG**acg**GGTC**a**TTCTAC**	3.79E-01
4.	miR-320a	PD-L1	1260	−13.16	**GG**ttg**AGAA**t**C**cctaattt**GA**g**AAGG**t **CC**--t**TCTT**g**G-**ccctt**CT**c**TTCC**g	3.10E-02

The bold and capital letters denote the complementary binding sites of miRNA on PD-L1. The binding sites are predicted from RNA22 software.

Moreover, CCK-8 assay results indicated a significant suppression (at 72 and 96 h, *p* < 0.01) of A549 and LLC (Lewis Lung Carcinoma) cells in the miR-4458 mimic group, showing that higher levels of miR-4458 could prevent cell proliferation [117]. Next, in female C57BL/6 xenograft LLC mice, the miR-4458 mimic group exhibited a significant reduction in tumor volume and weight compared to the miR-4458 negative control group. These data suggest that miR-4458 overexpression may inhibit NSCLC tumor growth.

Furthermore, flow cytometry results showed upregulation in the expression of immune cells such as CD8^+^ T-cells and CD4^+^ T-cells in the miR-4458 mimic group [117]. They also found increased IFN-γ and IL-2 serum levels in the miR-4458 mimic group, in stark contrast with decreasing IL-10 serum in the same group. IL-10 usually inhibits the activity of immune cells, whereas IL-2 increases the growth and proliferation of immune cells. IFN-γ activates the immune cells, so increased levels of IL-2 and IFN-γ enhance anti-tumor immunity.

STAT3 was predicted to be a potential target of miR-4458 using the TargetScan 6.2 database and was later verified by the luciferase reporter assay [117]. Moreover, a potential negative correlation was found between miR-4458 and oncogenic STAT3 gene expression (*F* = −0.201, *p* < 0.05) in 25 pairs of NSCLC tissues compared to normal lung tissue samples. It is demonstrated that STAT3 inhibits cell proliferation through a high expression level of miR-4458. Additional results indicate that miR-4458 mimics and inhibits STAT3 expression. Moreover, the CCK-8 assay confirms enhanced cell proliferation in A549 and LLC cells with the overexpression of STAT3.

**Fig. 6:**
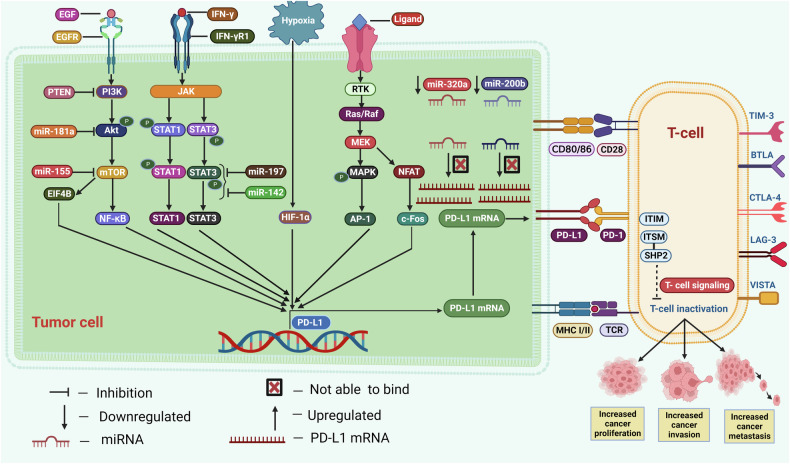
miRNA-mediated modulation of immune check points in tumor cells, ultimately leading to T-cell inactivation and resulting in immune escape.

To better understand the relationship between miR-4458 and STAT3 in regulating PD-L1 expression, the authors found that miR-4458 overexpression downregulated the expression of PD-L1 (Fig. 6), but this downregulated was mitigated by STAT3 overexpression in A549 and LLC cells [117]. These results were also validated in xenograft LLC mice. The authors thus concluded that PD-L1 expression is negatively regulated by miR-4458 through STAT3.

The authors also evaluated the effects of manipulating the miR-4458/STAT3/PD-L1 axis on anti-tumor immunity in xenograft LLC mice [117]. They showed that STAT3 overexpression promoted tumor growth and was able to partially negate the inhibitory effects of miR-4458 overexpression on tumor growth, whereas PD-L1 knockdown was able to neutralize the growth-promoting effects of STAT3 overexpression. Additionally, flow cytometry analysis revealed that miR-4458 overexpression resulted in heightened levels of CD4^+^, CD8^+^, PD-1^+^ cells, as well as increased levels of serum IFN-γ and IL-2 levels coupled with decreased IL-10 levels. [117].

Overall, this study demonstrated that miR-4458 increases anti-tumor immunity in NSCLC by targeting STAT3 to downregulate PD-L1 expression. Thus, the miR-4458/STAT3/PD-L1 axis presents as an attractive target to improve ICI response in NSCLC.

### miR-526b-3p

miR-526b-3p is another microRNA that has been involved in immune responses and cancer. Studies have shown that miR-526b-3p can modulate immune responses by targeting various genes involved in immune cell activation and differentiation. For example, miR-526b-3p targets the chemokine receptor CXCR4 gene in T-cells, reducing T-cell migration and activation. Moreover, miR-526b-3p decreases tumor growth in a variety of malignancies such as colon cancer [118], NSCLC [119], and act as prognostic biomarker for glioma [120].

To establish the clinical potential of miR-526b-3p, Chen et al. demonstrated a decreased expression of miR-526b-3p in PC-9 and A549 lung cancer cell lines treated with 0.1 µg/ml cisplatin (CDDP) compared to CDDP-sensitive cell lines of the same origin [121]. qRT-PCR results further revealed that miR-526b-3p levels decreased ~2.38 fold in PC-9 and ~2.5 fold in A549 lung cancer cell lines compared to the control cell line BEAS-2B. Furthermore, miR-526b-3p mimics in CDDP-resistant cells reduce IC50 values in the miR-526b-3p group against the vector group. Similarly, ectopic expression of miR-526b-3p reduces cell survival, but its inhibitors increase cell survival. Using dual luciferase activity, researchers discovered that miR-526b-3p detrimentally controls the amount of STAT3 in lung cancer cells. Furthermore, by combining the 3’ UTR of STAT3 with miR-526b-3p or miR-526b-3p mutant reduces the miR-526b-3p groups compared to the control group.

Furthermore, STAT3 expresses ~1.45 fold higher in CDDP-resistant lung cancer cell lines than CDDP-sensitive lung cancer cell lines. Exogenous overexpression of STAT3 reduces miR-526b-3p regulation during cell growth. Therefore, it suggests that miR-526b-3p suppresses STAT3 in CDDP-resistant lung cancer.

Likewise, miR-526b-3p reduces the expression of PD-L1 in lung cancer cells. The predicted binding site on PD-L1 mRNA by miR-526b-3p is shown in Table 2. Given that the activation of PD-1/PD-L1 improves immune evasion in cancer by reducing CD8^+^ T-cells, it is unclear how the introduction of miR-526b-3p affects the CD8^+^ T-cell population. Flow cytometric analysis revealed that STAT3 prevents the promotion of CD8^+^ T-cells, while miR-526b-3p enhances their population.

In concordance with the abovementioned results, STAT3 increases PD-L1 expression, which leads to chemoresistance in NSCLC. Furthermore, the suppression of miR-526b-3p increased the expression of STAT3 and PD-L1 (Fig. 6).

The authors further demonstrated that in the presence of an immunotherapeutic drug Avelumab, the level of PD-L1 expression decreases without significantly affecting the expression levels of STAT3 or miR-526b-3p. Additionally, the CD8^+^ T-cell counts decreased in the miR-526b-3p inhibitor-treated group. These results suggest that miR-526b-3p inhibitor increased cell migration while Avelumab impedes cell motility.

Likewise, the authors showed that miR-526b-3p mediates anti-tumor effects and that its inhibition was neutralized with Avelumab treatment, indicating that PD-L1 is a downstream target of miR-526b-3p/STAT3 in lung cancer. Also, transwell assay results suggest that overexpression of miR-526b-3p suppresses T-cell migration, while its knockdown enhances T-cell migration. Besides, the PD-L1 expression decreased about ~1.25-fold in the overexpressing xenograft mouse model of miR-526b-3p compared to the control mouse group. Based on the above data, we can infer that miR-526b-3p can modulate the expression of PD-L1 and STAT3 in cancer cells and inhibit cell proliferation, migration, and CDDP resistance [121].

These findings suggest that miR-526b-3p may have therapeutic potential in cancer immunotherapy by modulating immune responses and targeting critical regulators of tumor growth and immune evasion. Further research is necessary to deeply comprehend the clinical uses of miR-526b-3p in immunotherapy for lung cancer.

### miR-34 family

There are three members in the miR-34 family, specifically miR-34a, miR-34b, and miR-34c. Among all miR-34 family members, miR-34a expression was higher than miR-34b and miR-34c in the HCT116, H460, and H1299 NSCLC cell lines. Clinical investigations have shown an inverse association between miR-34 family expression and survival in cancer [122].

To elucidate the function of the miR-34 family in the context of immunotherapy, Cortez et al. [122] observed that miR-34 acts as an inhibitor of PD-L1 in NSCLC cells and tumor tissues[122]. Additionally, they discovered a putative miR-34 binding site in the 3’ untranslated region of the PD-L1 mRNA at positions 932-938, which further supports their theory. Moreover, exogenous overexpression of miR-34a leads to decreased PD-L1 protein expression compared to the scrambled control, indicating a negative regulation between miR-34 and PD-L1 (Fig. 6).

Further, to investigate the consequence of miR-34a expression on PD-L1 expression in a syngeneic mouse model of NSCLC, MRX34, a liposomal nanoparticle laden with miR-34a mimics, was subcutaneously injected. Further, qRT-PCR and western blotting results exhibited increased levels of miR-34a in tumors while concurrently downregulating tumor PD-L1 mRNA and PD-L1 protein. Flow cytometry confirmed the miR-34-induced repression of PD-L1, as PD-L1 expression was significantly lower in the MRX34 group than in the control group (mean PD-L1 expression percentage of the control group (30.4% vs 42.9%)), which is also supported by IHC finding. The liposomal administration of miR-34 mimics also suppressed PD-L1 expression in NSCLC xenografts mice. In addition, an in vitro study demonstrated that miR-34 mimics significantly reduced (~70%) luciferase reporter expression. Later, a multidose efficacy investigation was also carried out in the syngeneic mouse paradigm to examine the impact of MRX34 on cancer development, the tumor microenvironment, and its associated immune cells. MRX34 mimics enhance the fraction of CD8^+^ cells in tumors while decreases the amount of tumor-infiltrating PD-1^+^ T-cells and macrophages. CD8^+^ T-cells spiked much more in MRX34 and radiation treatment (XRT) combination than in either therapy alone (44.2% Vs 26.1%). Furthermore, in the combination condition, MRX34 inhibited the effects of XRT on macrophages and Tregs compared to XRT alone. The TNF-α and IFN-γ also increased in combination therapy which seems much more effective.

Mechanistically, to find how PD-L1 is affected by p53, three systems are used: first being the isogenic HCT116 p53−/− and p53+/+ colon cancer cells treated with the p53 stabilizer nutlin 3, second being the p53-inducible H1299 lung cancer cells treated with ponasterone A (PoA) and third being the H460 lung cancer cells transfected with a p53-specific or a scrambled shRNA. MiR-34a, miR-34b, and miR-34c expression levels were also considerably higher in wild-type p53-expressing cells (HCT116 p53+/+ and p53-inducible H1299 treated with PoA) than in untreated cells (HCT116 p53−/− and p53-inducible H1299). However, PD-L1 was deleted or expressed at lower levels in cells expressing wild-type p53, demonstrating that activation of p53 favored PD-L1 downregulation relative to controls. To validate this negative relationship between miR-34a and PD-L1 expression in vivo, tissues from patients harboring p53-wt and p53-mutated (R175) NSCLC were employed. The results revealed that compared to NSCLC tumors with wild-type p53, those with mutant p53 have low levels of miR-34a and high levels of PD-L1. Additionally, a significant inverse correlation (*r* = −0.29, *p* < 0.001) was observed between the expression of p53 and PD-L1. Further analysis comparing the expression of PD-L1 in NSCLC tumors with p53 mutations (n = 84) and p53 wild-type tumors (*n* = 97) showed that the latter had significantly higher levels of PD-L1 compared to the former (*P* = 0.03). Consequently, patients with p53 mutations exhibited lower levels of miR-34a compared to those with wild-type p53 (*p* = 0.01) [122].

These findings suggest that miR-34a regulates the evasion of tumors from the immune system through the p53 pathway by targeting PD-L1. However, the potential use of miR-34 in cancer therapy needs further investigation.

### miR-200b

The miR-200 family comprises five members, which are grouped into two chromosomal clusters (miR-200a/200b/429 and 200c/141). miR-200b maps to the long arm of chromosome 1q36.33 and has been implicated in several types of cancer and specifically targets transcription factors that promote EMT, such as zinc-finger E-box-binding homeobox 1 (ZEB1). It is downregulated in melanoma [123], esophageal squamous cell carcinoma [124], and hepatocellular carcinoma [125], but upregulated in colorectal cancer [126], prostate cancer [127] and breast cancer [128].

Recent studies conducted by Chen et al. (2014) revealed that the microRNA-200b (miR-200b) acts as a cell-autonomous antagonist of EMT and malignancy and targets PD-L1 [129]. The downregulation of miR-200b has been observed in lung cancer patients, indicating its potential role in cancer progression (Fig. 6).

Furthermore, to better understand the relationship between PD-L1, EMT potential, and the miR-200/ZEB1 axis, their expression was studied in lung cancer cell lines. The predicted binding site on PD-L1 mRNA by miR-200b is shown in Table 2. It was found that while PD-L1 expression was elevated in quasi-epithelial cells (murine 393P and human HCC827) with persistent ZEB1 expression, it was elevated in mesenchymal lung cancer cell lines (human H157, H1155, H1299, and H460; murine 344SQ, 531LN2, and 393P ZEB1). A strong EMT inducer, transforming growth factor-, disrupt the normal ZEB1-miR-200 double-negative feedback loop and produced a mesenchymal phenotype.

Transforming growth factor-β increased the levels of ZEB1 and PD-L1 in both human and mouse lung cancer cells. Previous studies have shown that IFN-γ increases the expression of PD-L1 in natural killer cells, T-cells, and macrophages. In a co-culture environment, it was found that IFN-γ stimulation increased the expression of PD-L1 in tumor cells. Mesenchymal tumor cells (344SQ and 393P ZEB1) were more sensitive to IFN-γ than epithelial tumor cells (344SQ miR-200 and 393P). These results indicate that the miR-200/ZEB1 axis controls the production of PD-L1 by tumor cells in the presence or absence of IFN-γ. Additionally, it was found that the miR-200 family members directly regulate PD-L1, as demonstrated by the inhibition of luciferase reporter activity when miR-200b or -200c pre-miRs were co-transfected into murine (344SQ) or human (H157 or H1299) lung cancer cells after transfection of wild-type (WT) PD-L1 3′-UTR reporter construct.

These results unequivocally show that in the presence or absence of IFN-γ, the miR-200/ZEB1 axis controls the production of PD-L1 by tumor cells. When miR-200b or −200c pre-miRs were co-transfected into murine (344SQ) or human (H157 or H1299) lung cancer cells after transfection of wild-type (WT) PD-L1 3′-UTR reporter construct, luciferase reporter activity was inhibited, proving that the miR-200 family members directly regulate PD-L1.

Moreover, significantly fewer CD8^+^ TILs and a higher proportion of weary CD8^+^ T-cells were found in 393P cell tumors that expressed ZEB1. It was discovered that the Kras/p53 (KP) mice, in which miR-200 loss induces EMT and metastasis, had considerably fewer CD8^+^ T-cells in their lung tissues than genetically modified K-rasLA1 mice, which form non-metastatic lung adenocarcinomas. There were significantly fewer total CD8^+^ TILs and a higher proportion of weary CD8^+^ T-cells in 393P tumors expressing ZEB1. It is further suggested that miR-200 downregulation enhances the expression of PD-L1 on tumor cells, which promotes CD8^+^ T-cell dampening in the tumor microenvironment and promotes metastasis [129].

IFN-γ has been the subject of much research, and it is well-known that this cytokine is essential for tumor surveillance. It is a potent master regulator of PD-L1 expression, which was suggested to be a factor in the immune evasion of tumors. Again, highlighting that mesenchymal tumors are more susceptible to IFN-γ, its inhibition significantly reduced fatigued T lymphocytes in mesenchymal tumors while having no meaningful effect on epithelial tumors. When seen as a whole, the data show that the miR-200/ZEB1 axis is a downstream regulator of PD-L1 that can control the outcomes of the IFN-γ pathway.

The involvement of the miR-200/ZEB1 axis was further examined in lung adenocarcinoma metastasis, and it was found it was discovered that ZEB1-induced decreases in miR-200 increase tumor cell invasion and metastasis. ZEB1 and miR-200 form a double-negative feedback loop that mediates the dynamic transition from epithelial to mesenchymal states. PD-L1 inhibition was observed to considerably increase CD8^+^ T-cell infiltration, reverse the tired T-cell phenotype, lessen tumor burden, and limit metastasis. However, modulating PD-L1 via the miR-200/ZEB1 axis may not entirely explain for the immunosuppressive effects identified [129].

Furthermore, it has been found that downregulation of miR-200 enhances the expression of PD-L1 on tumor cells, leading to CD8^+^ T-cell suppression in the tumor microenvironment and promoting metastasis. These studies suggest that miR-200b could be a potential therapeutic target for treating lung cancer. The negative correlation between miR-200b and PD-L1 expression suggests that miR-200b could be used as a therapeutic marker for lung cancer patients.

### miR-16-5p

miR-16-5p, which is present at 13q14.3 is produced by *MIR16-1* gene [130]. It plays a significant role in the emergence of various cancers [130]. miR-16-5p is downregulated in breast cancer [131] whereas in lung cancer via NF-kB signaling [132].

Chen et al. (2022) identified the presence of PD-L1 protein in fresh lung adenocarcinoma (LUAD) tissues, where the authors found thirty-two out of sixty patients were positive for PD-L1 expression [133]. Additionally, the level of PD-L1 was associated with the T-stage (tumor size stage) in PD-L1-positive LUAD patients. Specifically, the patients at T3 stage had higher PD-L1 expression compared to patients at the T1 stage (*p* < 0.01). Moreover, PD-L1 levels decreased following PD-L1 inhibitor therapy.

Further to assess the expression of exosomal miR-16-5p in LUAD patients, it was observed that miR-16-5p expresses ~2.94 fold significantly lower than those in healthy controls (*p* < 0.01). When compared to patients with tumor sizes at the T2 with T1, the T2 stage patients displayed higher PD-L1 expression. After treating PD-L1-positive LUAD patients with PD-L1 inhibitors for 15 weeks, the expression of miR-16-5p derived from serum exosomes significantly increased (*p* < 0.05). Interestingly, following 15 weeks of PD-L1 inhibitor treatment, variations in exosomal miR-16-5p expression were shown to have a negative relationship with PD-L1 protein levels in tissues (*p* < 0.05) (*r*^2^ = 0.2077). According to follow-up studies, LUAD patients with low serum exosomal miR-16-5p levels had more significant therapeutic effects and overall survival than those with higher levels. Likewise, compared to BEAS-2B (control) cell culture medium, the exosomal miR-16-5p levels in A549 (~2.27 fold) /PC9 (~2.5 fold) /HCC827 (~2.38-fold) cell culture media were downregulated (*p* < 0.05 or *p* < 0.01).

Furthermore, to assess the effect of miR-16-5p in HCC827/PC9 cells and by transfecting miR-16-5p mimic, the results revealed that miR-16-5p was found to be ~2.2-fold higher in exosomes in the cell culture medium (*p* < 0.01) (Fig. 6).

Transfection of HCC827 cells with a pcDNA-PD-L1 vector resulted in PD-L1 overexpression (*p* < 0.01), which led to a reduction in exosomal miR-16-5p in the HCC827 cell culture medium (*p* < 0.05). The findings revealed that overexpression of exosomal miR-16-5p in cell culture medium decreased the role of PD-L1 overexpression (*p* < 0.05), which was associated with increased tumor volume and weight compared to the NC vector group. The results showed that the pcDNA-PD-L1 group had higher levels of PD-L1 protein than the NC vector group.

The primary takeaways from this study’s findings were that the expression of exosomal miR-16-5p and tissular PD-L1 was related to both T-stage (tumor size stage) and the therapeutic benefit of PD-L1 inhibitors in PD-L1-positive LUAD patients. Thus, the results suggest that serum exosomal miR-16-5p could be a potential biomarker for PD-L1 inhibitor-dependent immunotherapy and a tumor suppressor in LUAD [133].

### miR-320a

miR-320a present on 8p21.3 primarily downregulated in various cancers like ovarian cancer via targeting SMG1 [134], in NSCLC [21], in bladder carcinoma by targeting ITGB3 [135] in glioma by targeting SND1 and β-catenin [136] but upregulated in prostate cancer by negatively regulating TP73-AS1 lncRNA [137].

To analyse the regulatory mechanism of miR-320a in immunotherapy, Dong et al. [138] injected Diethylnitrosamine (dose range—25 µg/g of body weight), a chemical carcinogen, into an intraperitoneal cavity to induce lung cancers. This chemical mutagen can trigger mutations, and the progression of lung cancer is mainly determined by the incubation period. After 6 months of incubation, Diethylnitrosamine can initiate early lung cancer development. However, 9 months period leads to advanced lung cancer development, confirmed by histopathological examination [138].

In situ hybridization results revealed that miR-320a has higher expression in initial lung cancer tissue compared to advanced lung cancer stages. It was selectively inhibited by complementary binding with its guide strand. LNA-anti-miR320a oligonucleotide (5′-uscsgcccucucaacccagcusususus-Chol-3′) was used to block miR-320a. As a result, the complexity of advanced lung cancer increased. Furthermore, it was determined that due to the miR-320a blockade, the PD-L1 level increased. With this, it can be concluded that miR-320a is downregulated in the advanced lung cancer stage, and miR-320a levels negatively correlate with PD-L1 levels, suggesting the tumor-suppressive roles of miR-320a (Fig. 6). The predicted binding site on PD-L1 mRNA by miR-320a is shown in Table 2.

PD-L1 is minimally expressed in control tissue, but it is subsequently overexpressed in early-stage and advanced lung cancer, increasing its level by 2.2 and 5.6 folds, respectively. In contrast to the unblocked controls, PD-L1 is more prevalent in tissue where miR-320a is inhibited. PD-L1 is raised 1.6 and 1.9 times higher in initial and advanced miR-320a blocked lung tissue than in initial and advanced miR-320a non-blocked tissues [138].

From the abovementioned results, we can conclude that miR-320a has the potential to serve as a diagnostic biomarker for lung cancer, as its reduced expression correlates with PD-L1 overexpression in both the early and advanced stages of the disease.

### miR-4315

To investigate the relationship between T-cells and anti-PD-1 therapy, Guyon et al. looked into whether exposing T-cells to anti-PD-1 treatment may increase the production of exosomal miRNA [139]. Although molecular mechanisms are still unclear, exposure of T-cells to anti-PD-1 treatment increased exosomal miRNA-4315 levels.

According to the miRGen.v3 program’s predictive study, the expression of five FoxO1-regulated miRNAs (miR-101-5p, miR-612, miR-3671, miR-4315, and miR-let7i) was examined. These five miRNAs are expressed in T-cells. Surprisingly exosomes obtained from T-cells exposed to PD-1 contained ten times more miR-4315 than exosomes derived from T-cells exposed to the IgG control.

Considering the previous evidence demonstrating that exosomal miR-4315 restricts apoptosis, it is speculated that miRNA-4315 can target a Bim, a proapoptotic protein in the BCL2 family. A mimic of miR-4315 reduced luciferase activity coupled to the 3′UTR/Bim plasmid and downregulated both protein and mRNA levels of Bim in cells. Anti-miR-4315 remarkably prevented Exo/PD-1 from further reducing the expression of Bim in A172 cells and was co-immunoprecipitated with 3′UTR/Bim.

Similar studies have been conducted on the lung cancer cell line A549, (the ovarian cancer cell line OV90), and the breast cancer cell line MCF7. Exo/PD-1 attenuated the cell death driven on by each treatment, and as was discovered with the A172 glioma cell line, these effects were blocked by an anti-miR-4315. As expected, the downregulation of Bim expression and the reduction of PARP and Caspase-3 cleavage were associated with cell death inhibition. The study revealed that exomiR-4315 derived from T-cells exposed to PD-1 inhibited Bim expression. Resistance to chemotherapy was enhanced as a result of the downregulation of apoptosis produced by the integration of miR-4315 into cancer cell lines.

Further, the effectiveness of exo miR-4315 as a biomarker was investigated. It was discovered that in patients with lung cancer undergoing anti-PD-1 therapy, the changes in exomiR-4315 expression over time are correlated with a serum biomarker associated with resistance to apoptosis. In four lung cancer patients receiving PD-1 treatment, exomiR-4315 levels and serum cytochrome c levels were measured to determine the therapeutic relevance. Additionally, Pearson’s correlation analysis showed that serum cytochrome c concentrations and longitudinal exomiR-4315 expression were associated significantly in each patient recruited. Overall, these findings indicate that in lung cancer patients receiving PD-1, exomiR-4315 expression was shown to be inversely linked with a serum biomarker for apoptosis resistance.

The effect of a “Bim/BH3 mimic medication” such as ABT263, on CDDP cisplatin resistance in A549 cells exposed to Exo/PD-1 was then studied, taking into consideration the evidence presented above relating the ineffectiveness of CDDPcisplatin + PD-1 treatment to exomiR-4315-induced Bim downregulation. A549-inoculated xenograft mice were used to evaluate the effectiveness of ABT263. As anticipated, CIS therapy reduced the number of tumors brought on by A549 [139].

Overall, the results suggest that ABT263 can reverse the resistance induced by Exo/PD-1 to CDDP treatment. Moreover, there was a significant inverse correlation (*r* = −0.9566; *p* = 0.0028) between the treatment’s effect on cancer and the level of serum cytochrome c. These findings highlight the potential of exomiR-4315 as a biomarker for assessing the efficacy of miR-4315 as an anti-cancer therapy (Fig. 6).

### miR-181a

Recent investigations have revealed that distinct miRNAs express in various human malignancies. miR-181a is upregulated in breast cancer via regulating NDRG2 levels [140], also upregulated in thyroid cancer [141], downregulated in prostate cancer by affecting cell cycles [142], and downregulated in ESCC via affecting the MAPK pathway [143].

To investigate the relationship between miR-181a and PD-L1 in CDDP-resistant NSCLC, Chen et al. [133] showed that A549 cells treated with 10 µg/ml CDDP for 24 h exhibited an increase in PD-L1 expression by ~2.3-fold. Similar results were observed in another NSCLC cell line, H69, when treated with CDDP. In A549R cells, which were developed to be resistant to CDDP, PD-L1 expression was already ~2-fold higher compared to untreated A549 cells, and additional CDDP treatment did not significantly affect PD-L1 expression [144].

To investigate the link between miR-181a and PD-L1 expression in NSCLC, miR-181a was transiently overexpressed in CDDP-treated A549R cells, which resulted in a significant reduction in PD-L1 mRNA levels. This suggests that PD-L1 expression in NSCLC is negatively regulated by CDDP-induced miR-181a and that higher PD-L1 expression in CDDP-resistant NSCLC cells may be associated with lower levels of miR-181a **(**Fig. 6**)**.

Because tumoral PD-L1 can limit cytotoxic CD8^+^ T-cell activity, cause T-cell fatigue, and promote tumor formation, mice were injected with LL/2, an immunogenic lung malignancy, and the tumor growth curve and T-cell function were examined. Exogenous miR-181a significantly inhibited the development of LL/2 tumor cells in vivo compared to control LL/2 cells. Lower PD-L1 expression was also observed in these cells. Additionally, TILs were also examined to measure cytotoxic CD8^+^ T-cell activity. Exhausted T-cells, defined as PD-1^+^ and Tim-3^+^ PD-1^+^ CD8^+^ T-cells, were significantly reduced in the TIL of miR-181a-transfected LL/2 cells. Both TNF-α^+^ CD8^+^ TILs and (Granzyme B) GZMB^+^ CD8^+^ TILs were also found to be significantly higher compared to the control arm [144]. These results suggest that increasing miR-181a expression in lung cancer may boost anti-tumor responses and prevent T-cell exhaustion, thereby highlighting miR-181a as a potential target of miRNA mimic therapy in lung cancer.

## Role of PD-1/PD-L1 immune checkpoint inhibitors in lung cancer

The notion of antibody immunotherapy targeting evolved from exploiting the expression of inhibitory checkpoint molecules on the surface of cancer cells. Inhibiting PD-1 and PD-L1 interactions activate the suppressed immune system activity while disabling the tumor’s “immune shielding,” which enhances the anti-tumor effect [145].

FDA-approved antibody medications have revolutionized the cancer immunotherapy field [146] (Table 3). In recent years, the utilization of immune checkpoint inhibitors (ICIs), specifically anti-PD-1/PD-L1 immunotherapy, has demonstrated encouraging outcomes in the clinical setting. These therapies have been successful in promoting tumor regression and inhibiting metastasis. In this section, we briefly discuss the mechanisms of action of each of these ICIs, and their performance in recent clinical trials involving lung cancer patients.

**Table 3:** Major PD-1/PD-L1 immune checkpoint inhibitors used in clinical practices to treat lung cancer.

Sr.No.	Drug	Trademark	FDA approval date	Acting domain	Major binding sites	Type of antibody	Affinity (K_D_)	Population	Stage	Overall response rate (ORR)	References
1.	Nivolumab	Opdivo	Sept., 2014	N-loop; FG, BC loop	D29, R30, S60, K131;P28, L128, A129 etc.	IgG4/anti PD-1	1.45 nM	France, Germany, Italy, and USA	stage IIIB or IV	17%	[70]
2.	Pembrolizumab	Keytruda	Dec., 2014	C’D loop	N66, T76, K78,S87,K131; F63, E84, S87N etc.	IgG4/anti PD-1	400 pM	Japan	stage IV	40%	[152]
3.	Atezolizumab	Tecentriq	May, 2016	CC’FG antiparallel -sheet; BC, CC’, C’C” and FG loops	E58,Q66, V111 (hydrogen bond); R113, R125 (salt bridge)	IgG1/anti PD-L1	1.75 nM	China	stage IV	11.90%	[157]
4.	Durvalumab	Imfinzi	May, 2017	Parallel -sheet; A, G, F and CC’ loops/strands	D26, R113;NY123, K124, R125 (hydrogen bond)	IgG1/anti PD-L1	0.667 nM	Asia, Australia, Europe, North America, South America, and South Africa.	stage III	66.30%	[160]
5.	Avelumab	Bavencio	March, 2017	Parallel -sheet; CC’FG loop/strand	I54, Y56, M115, Y123 (hydrogen bond); E58, Q66, R113, A121	IgG1/anti PD-L1	0.046 nM	USA	stage IIIB or IV	12%	[64]

## PD-1 inhibitors

### Nivolumab

Nivolumab, whose brand name is Opdivo, was first approved by FDA in 2015 to treat patients with lung cancer. Later, it was proven to be safe and effective for the treatment of other cancers, such as melanoma and renal cell carcinoma [64]. Nivolumab is an IgG4 antibody-based immune checkpoint inhibitor that binds to the PD-1 receptor on activated T-cells [147]. Mechanistically, it binds to the flexible loops of PD-1, which serves as the primary interface between the antibody and PD-1 [148]. Moreover, complementarity-determining regions (CDRs) of the light chain of the nivolumab antibody bind to the FG loop of PD-1 [69, 146, 149]. This interaction reduces the affinity between PD-1 and PD-L1, consequently enhancing the host’s immune response against tumors [70]. Additionally, nivolumab alleviates the immune suppression of activated tumor-specific T-cells through its binding to PD-1, enabling these cells to perform their cytotoxic functions [70].

Regarding the efficacy of nivolumab in stage IV NSCLC patients, Gettinger et al. recruited 129 patients with a median age of 65 years. Their study revealed that treatment with nivolumab resulted in an objective response in 22 patients (17%). This group of respondents displayed a median progression-free survival (PFS) of 17.0 months (95% CI, 11.0–24.7). Moreover, overall survival (OS) rates for this group were 42% (95% CI, 33–50) at 1 year, 24% (95% CI, 17–33) at 2 years, and 18% (11–25) at 3 years [150].

In another study, 358 resectable NSCLC patients with stage IB and IIIA were enrolled. Among these patients, 179 received neoadjuvant platinum-based chemotherapy alone, while the remaining 179 received neoadjuvant nivolumab plus platinum-based chemotherapy. The authors observed that patients who received nivolumab plus chemotherapy had a median event-free survival of 31.6 months (95% CI, 30.2 to not reached) [151]. In contrast, those treated with chemotherapy alone had a lower median event-free survival of 20.8 months (95% CI, 14.0 to 26.7) (hazard ratio for disease progression, disease recurrence or death, 0.63; 97.38% CI, 0.43–0.91, *P* = 0.005). Additionally, nivolumab plus chemotherapy outperformed chemotherapy alone in event-free survival and pathological complete response across all patient subgroups including PD-L1 expression level and type of platinum therapy (CDDP or carboplatin). Hence, combining neoadjuvant nivolumab with chemotherapy significantly improved pathological complete response and event-free survival in patients with resectable NSCLC compared to chemotherapy alone.

In a separate study, Ready et al. investigated the efficacy of nivolumab standalone or in combination with ipilimumab in SCLC patients with metastatic solid malignancies. The study demonstrated that the patients who received nivolumab plus ipilimumab had improved overall response rates compared to those receiving nivolumab alone (21.9% vs. 11.6%; odds ratio: 2.12; 95% CI, 1.06–4.26, *p* = 0.03). Furthermore, the median OS was 5.7 months (95% CI, 3.8–7.6) as compared to 4.7 months for nivolumab alone (95% CI, 3.1–8.3) [152].

### Pembrolizumab

Pembrolizumab is a humanized IgG4 anti-PD-1 antibody (mAb)that was first approval by the FDA in 2015 for advanced NSCLC treatment [64, 153]. The interaction between pembrolizumab and PD-1 involves two important sites within the PD-1 protein: the C′D loop (site I) and the C, C′, and F strands (site II) [154]. Site I imparts affinity to pembrolizumab while site II is directly involved in the PD-1/pembrolizumab interaction. [69].

According to a study by Swami et al. which involved giving a niraparib plus pembrolizumab combination regime to stage IV lung cancer patients, PD-1 inhibitors and poly (ADP-ribose) polymerase (PARP) inhibitors may synergize to boost innate and adaptive anticancer immune responses. The results revealed that out of two cohorts, the cohort with PD-L1 TPS (tumor proportion scores) ≥ 50%) (cohort 1) displayed an ORR of 56.3% (95% CI, 29.9–80.2%) [155]. The verified ORR for cohort 2 (PD-L1 TPS 1–49%) was 20.0% (95% CI, 5.7–43.7%); 4 of the 20 patients had partial responses (PRs), and four patients experienced disease progression. Disease control rates (DCR) for cohorts 1 and 2 were 87.5% (95% CI, 61.7–98.4%) and 70.0% (95% CI, 45.7–88.1%), respectively. In conclusion, Niraparib and pembrolizumab exhibited clinical activity in patients with advanced or metastatic NSCLC, in which 56.3% of patients responded to the combination therapy.

In a study conducted by Grassino et al., it was found that the combination of pembrolizumab, pemetrexed, and carboplatin/CDDP led to a significantly longer overall survival (OS) compared to the placebo plus pemetrexed-platinum group(hazard ratio, 0.49, 95% CI, 0.38–0.64, *P* < 0.001).Additionally, the pembrolizumab combination therapy demonstrated improved PFS with an HR of 0.52 (95% CI, 0.43–0.64, *P* < 0.001), specifically in previously untreated metastatic NSCLC patients without EGFR/ALK alterations. Five-year OS rates were 19.4% compared to 11.3%, while 5-year PFS rates were 7.5% compared to 0.6%. Also, ORR (95% CI) values were 48.3% (43.4 to 53.2) and 19.9% (14.7 to 26.0), respectively. In the pembrolizumab plus pemetrexed-platinum group, the median (95% CI) PFS2 was 17.0 (15.0–19.2) months, compared to 9.1 (7.6–10.8) months in the placebo plus pemetrexed-platinum group (HR, 0.54; 95% CI). In comparison to 7.8% (4.7–12.1), 5-year PFS2 rates (95% CI) were 16.7%. With a 95% confidence interval, 5-year PFS2 rates were 16.7% compared to 7.8%. Consequently, compared to the placebo plus pemetrexed-platinum group, patients in the pembrolizumab plus pemetrexed-platinum group continued to show extended survival and sustained anticancer activity after 5 years. Based on these results, the combination of first-line pembrolizumab plus a platinum drug and pemetrexed is the recommended course of treatment for these patients [156].

Similarly, another study assessed the efficacy and safety of pembrolizumab in patients with metastatic NSCLC who have received prior first-line immunotherapy (PD-1/PD-L1 inhibitor alone or in combination with platinum-doublet chemotherapy). The topline analysis revealed that the median OS was 12.3 months versus 12.1 months (*p* = 0.0643), the HR value was 0.682 (95% CI, 0.454–1.025, *P* = 0.062), and the 2-year OS rate was 35.82% versus 11.88% in a total of 129 patients with ICIs exposure (plinabulin + docetaxel vs. docetaxel alone). These findings indicate that plinabulin exhibits good immune-oncology capabilities when combined with ICIs.In conclusion, this therapy combo holds promise in overcoming ICIs resistance and offering a new second-line therapeutic option for patients with advanced NSCLC who have already received immunotherapy [157].

## PD-L1 inhibitors

### Atezolizumab

The human IgG1 monoclonal antibody atezolizumab, or MPDL3280A or Tecentriq, was the first PD-L1 inhibitor to be approved by the FDA for urothelial cancer in May 2016. Atezolizumab binds to PD-L1 on tumor surfaces, preventing its interaction with PD-1 and resulting in an anti-tumor effect [23]. Its genetically engineered Fc fragment avoids the antibody-dependent cell-mediated cytotoxicity (ADCC) effect [158]. Several studies have shown the significant immunotherapeutic impacts of atezolizumab in various cancers, including kidney cancer, bladder transitional cell carcinoma, and breast cancer [159].

Recently, Herbst et al. [160] observed that patients with metastatic nonsquamous or squamous NSCLC who had wild-type EGFR and ALK tumors with high PD-L1 expression had a median OS of 20.2 months when given atezolizumab compared to 13.1 months with platinum-based chemotherapy alone. Also, within the subgroup of patients with a high blood-based tumor mutational load, atezolizumab was more advantageous in terms of overall and progression-free survival. The conclusion reflects that patients with NSCLC who had high levels of PD-L1 expression had substantially longer overall survival after receiving atezolizumab treatment compared to those that received platinum-based chemotherapy [160].

Another study by Seto et al., investigated the therapeutic effects of combining bevacizumab with atezolizumab at a fixed dosage for advanced NSCLC patients with PD-L1 expression exceeding 50% but without EGFR/ALK/ROS1 alterations. Of the 39 included patients, 36 (92.3%) had a smoking history, and 33 (84.6%) had stage IV NSCLC. The 12-month progression-free survival (PFS) rate was 54.9% (95% CI, 35.65–70.60), and the overall survival (OS) rate was 70.6% (95% CI, 50.53–83.74). The study concluded that the combination of atezolizumab and bevacizumab showed promise as a therapy for NSCLC with high PD-L1 expression [161].

### Durvalumab

Durvalumab, a humanized mAb, binds with high affinity to PD-L1 and inhibits PD-L1 and PD-1 interaction on T-cells [162]. Park et al. examined the efficacy of durvalumab in 51 small-cell lung carcinoma patients. They found that there was no significant difference in the hazard ratio for PFS and OS between patients with high PD-L1 expression and those with low PD-L1 expression (PFS hazard ratio: 0.70; 95% confidence interval: 0.31–1.58; OS hazard ratio: 0.64; 95% confidence interval: 0.22–1.84). Moreover, the median PFS was 14.4 months (95% confidence interval), the 24-month PFS rate was 42.0%, and the median follow-up time was 26.6 months. A 24-month overall survival rate of 67.8% did not exceed the median overall survival [163].

Similarly, Cheng et al. evaluated first-line durvalumab plus tremelimumab (DT) versus chemotherapy in metastatic NSCLC. They observed that patients with PD-L1 TC (tumor cell) expression <1% had an OS of 15 months (95% CI,10.5–27.4) when given durvalumab plus DT vs 11.7 months when given durvalumab plus chemotherapy. In addition, 24-month OS rates were 36.0 (95% CI,18.2–54.2%) vs 17.9% (95% CI,6.5–33.7) respectively. [164].

Johnson et al. evaluated whether the efficacy of durvalumab increases when given in combination with histone deacetylase (HDAC) inhibitors. Mocetinostat (class I/IV HDAC inhibitor) and durvalumab were given to sixty-three patients with advanced NSCLC across cohorts stratified by tumor PD-L1 expression and prior experience with ICI regimens. The cohorts’ ORR was 11.5%, and the median response time was 329 days. Patients with NSCLC who have previously received therapy with a checkpoint inhibitor showed clinical activity: ORR 23.1%. The most prevalent adverse treatment-related events were fatigue (41%), nausea (40%), and diarrhea (31%). The study concluded that the combination of durvalumab at the recommended dosage with mocetinostat was well tolerated. More importantly, the study showed that patients with NSCLC that were previously resistant to anti-PD-L1 treatment exhibited clinical activity [165].

### Avelumab

Avelumab, a human anti-PD-L1 IgG1 mAb, has been demonstrated to inhibit PD-L1, thereby reactivating T-cells. Additionally, through its natural Fc region, avelumab can trigger antibody-dependent cell-mediated cytotoxicity (ADCC). [166]. A study conducted by Corte et al. highlighted that avelumab, in combination with the anti-epidermal growth factor receptor (EGFR) drug cetuximab, led to cancer cell growth reduction in three-dimensional in vitro spheroid cultures made from NSCLC patient cells compared to single-agent treatment. When combined with cetuximab, avelumab increased clinical efficacy in six patients with PFS of at least 8 months. And out of three patients, one patient received therapy for 34 months, while the PFS for the other two patients was 15 and 19 months, respectively. Peripheral blood mononuclear cells serially collected data demonstrated long-term activation of NK cell-mediated ADCC. This suggests combining cetuximab and avelumab may be effective for specific NSCLC patients [167].

Similarly, Solomon et al. conducted a combinatorial study in which avelumab, axitinib, and palbociclib were administered to advanced NSCLC patients who had previously undergone at least two lines of treatment and showed no changes in EGFR, ALK, or ROS1. Out of 15 patients, PR were seen in four patients (27%) [PFS]: 14, 24, 25, and 144+ weeks). Eight out of 15 patients (53%) who received the combination of avelumab, axitinib, and palbociclib had a clinical benefit. This triplet was well tolerated and showed anticancer efficacy in NSCLC, including tumors that had progressed after pembrolizumab treatment [166].

In another study, Shafique et al. examined the effects of combining avelumab with pepinemab. The results indicated that the combination treatment exhibited a higher response rate compared to single-agent avelumab, particularly in the PD-L1 negative/low group. Among the 21 patients evaluated, five demonstrated PR, while four displayed clinical improvement after 1 year. The DCR was found to be 81%. Overall, the combination of pepinemab and avelumab was well-tolerated and proved to be effective in treating immunotherapy-resistant and PD-L1-negative/low NSCLC tumors.

## Conclusion, future perspectives, and limitations

The field of immunotherapy is captivating, and significant advancements have recently been achieved to deepen our understanding of how the host immune system influences tumor growth and treatment response. These developments have led to the discovery of novel immunological checkpoint inhibitors that have been approved for use in clinics. One of the most successful cancer drug discoveries in recent years is the development of immune checkpoint inhibitors. In 2011, the FDA approved the first immune checkpoint inhibitor (ipilimumab, an anti-CTLA-4 antibody), which marked a significant advancement in the field of immunotherapy as well as greatly enhancing the survival of NSCLC patients. Due to its tremendous success, ICIs have emerged as a fundamental therapeutic approach for various types of cancers.

Immune checkpoint molecules, which include inhibitory receptors or immunological checkpoints, play a vital role in maintaining self-tolerance and minimizing tissue damage caused by the immune system’s response to cancer. By inhibiting molecules such as PD-1 and PD-L1, immune checkpoint inhibitors can reactivate cytotoxic T-cells, enabling them to combat cancer. Additionally, co-stimulatory factors like CD28 enhance signaling when T-cell receptors detect antigens alongside the major histocompatibility complex (MHC). Recent studies have revealed that immune-inhibitory checkpoints like PD-1/PD-L1 and CTLA-4 play a significant role in the balance and evasion stages of cancer immune evasion. However, they also suppress the anti-tumor response when they bind to ligands on antigen-presenting cells (CTLA-4 binding to CD80/CD86) or tumor cells (PD-1 binding to PD-L1). Targeting and blocking these immune-inhibitory interactions using monoclonal antibodies (mAbs) have paved the way for a new generation of immunotherapy-based cancer treatments.

Innovative therapeutic methods including PD-1/PD-L1 inhibitors with other ICIs and DNA repair targeted medications are being tested in clinical trials. It is now predicted that cancer vaccines and cell-based chimeric antigen receptor T-cells (CAR T-cells) and combination immunotherapies are the future of lung cancer treatment.

This review article primarily focused on how PD-1/PD-L1 immunotherapy can be used to treat lung cancer in association with miRNAs. The rationale behind this paper was to explore more about immunotherapeutic in lung cancer and how non-coding RNAs like miRNA can help in combination with ICIs lung cancer treatment. However, during the literature analysis, we found that there are a lot of research gaps in this field of oncology, as it is also a new and emerging concept in the field of tumorigenesis. The main limitation of this study was that the research unveiled in this context is very narrow that needs to be divulged soon. And the reason behind the narrow study can be the less patient data because, usually patients are not willing to share their information with the researchers. Moreover, PD-L1 detection has a low accuracy value, so it is also problematic in the examination and treatment of patients. Also, different types of tumors at different sites may have different levels of PD-L1 expression. Another impediment was that there were a lot of research gaps in the papers. For example, none of the papers clearly defined the exact mechanism of how miRNAs interact with PD-1/PD-L1 protein and how they are interacting with each other to cure lung cancer. During the research, it was noticed that the latest research paper on this topic lacked the aforementioned facts. The particular sites or domains of miRNA and PD-1/PD-L1 interaction are not distinctly defined. Also, there are certain events that are related to immunotherapy treatment called as irAEs (immune-related adverse events) like colitis, gastrointestinal toxicity, neurotoxicity, and many other fatal phenomena. These may lead to some other diseases that may even worsen the condition. Moreover, the mechanistic studies on the action of drugs on the miRNA/PD-1/PD-L1 axis need to be unversed. So, there is a dire need to generate new therapeutic biomarkers that can provide relief to cancer patients in spite of harming the individual incongruously.
